# Enhancing glucose metabolism via gluconeogenesis is therapeutic in a zebrafish model of Dravet syndrome

**DOI:** 10.1093/braincomms/fcab004

**Published:** 2021-01-25

**Authors:** Rajeswari Banerji, Christopher Huynh, Francisco Figueroa, Matthew T Dinday, Scott C Baraban, Manisha Patel

**Affiliations:** 1 Skaggs School of Pharmacy and Pharmaceutical Sciences, University of Colorado, Anschutz Medical Campus, Aurora, Colorado, CA 80045, USA; 2 Department of Neurological Surgery, Epilepsy Research Laboratory, University of California, San Francisco, CA 94143, USA

**Keywords:** epilepsy, gluconeogenesis, metabolism, mitochondria, zebrafish

## Abstract

Energy-producing pathways are novel therapeutic targets for the treatment of neurodevelopmental disorders. Here, we focussed on correcting metabolic defects in a catastrophic paediatric epilepsy, Dravet syndrome which is caused by mutations in sodium channel NaV1.1 gene, *SCN1A*. We utilized a translatable zebrafish model of Dravet syndrome (*scn1lab*) which exhibits key characteristics of patients with Dravet syndrome and shows metabolic deficits accompanied by down-regulation of gluconeogenesis genes, *pck1* and *pck2*. Using a metabolism-based small library screen, we identified compounds that increased gluconeogenesis via up-regulation of *pck1* gene expression in *scn1lab* larvae. Treatment with PK11195, a *pck1* activator and a translocator protein ligand, normalized dys-regulated glucose levels, metabolic deficits, translocator protein expression and significantly decreased electrographic seizures in mutant larvae. Inhibition of *pck1* in wild-type larvae mimicked metabolic and behaviour defects observed in *scn1lab* mutants. Together, this suggests that correcting dys-regulated metabolic pathways can be therapeutic in neurodevelopmental disorders such as Dravet syndrome arising from ion channel dysfunction.

## Teaser

Banerji *et al.* identified a gluconeogenesis modulator, PK11195, a known mitochondrial translocator protein ligand that normalized metabolic deficits and suppressed electrographic seizures in a pre-clinical zebrafish model of Dravet syndrome. This suggests a novel metabolism-based therapeutic avenue to treat catastrophic paediatric epilepsies such as Dravet syndrome arising from ion channel mutations.

## Graphical Abstract

**Graphical Abstract:**
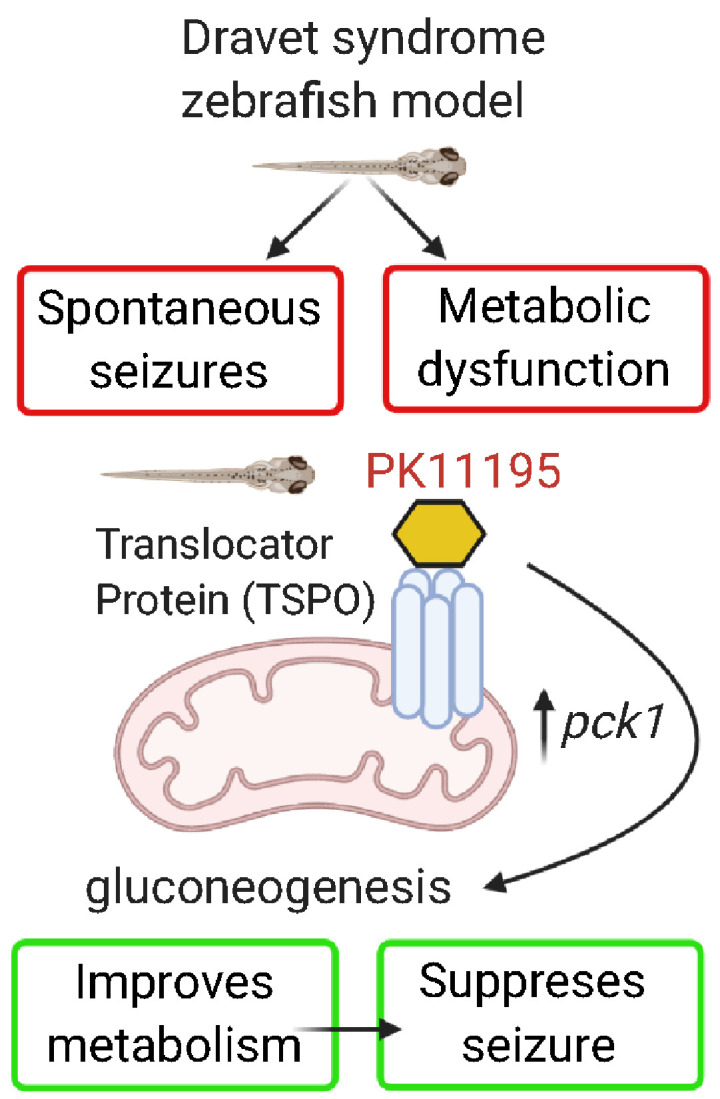

## Introduction

Epilepsy, regardless of its aetiology, is a heterogeneous group of disorders arising from altered ionic or synaptic transmission. It is a common neurological condition with a complex spectrum of disorders widely accepted as a disease of network excitability (England *et al.*, 2012; Pearson-Smith and Patel, 2017; Patel, 2018). While metabolic and bioenergetic alterations have been reported in acquired epilepsy syndromes, their role has remained secondary rather than central (Patel, 2018). In contrast, the role of metabolism has not been systematically investigated in genetic forms of epilepsy representing a major gap in our understanding of these catastrophic diseases. The vast majority of these genetic disorders arise from mutations in ion channels, signalling molecules or receptors (Porter *et al.*, 2012). Therefore, metabolism-based approaches to treat these disorders are a novel and promising alternative strategy. Dravet syndrome is a prototypical severe genetic form of epilepsy primarily affecting the paediatric population (Dravet, 2011). It is most commonly associated with *de novo* gene mutations in a voltage-gated sodium channel NaV1.1 gene, *SCN1A* (Depienne *et al.*, 2009; Catterall, 2010; Escayg and Goldin, 2010; Dravet and Oguni, 2013). Children affected with *SCN1A* loss-of-function mutations experience frequent prolonged spontaneous seizures, developmental delays, cognitive and behavioural deficits as well as increased risk of Sudden Unexpected Death in Epilepsy (Dravet, 2011; Li *et al.*, 2011; Skluzacek *et al.*, 2011; Dravet and Oguni, 2013; Kearney, 2013; Cooper *et al.*, 2016).

Glucose is the primary fuel required by the brain, the most metabolically demanding organ in the human body (Costantini *et al.*, 2008). Hypometabolism refers to a condition of decreased brain glucose consumption and has been reported in both acquired and genetic human epilepsies including Dravet syndrome (Ferrie *et al.*, 1996; Korinthenberg *et al.*, 2004; Benedek *et al.*, 2006; Craig *et al.*, 2012). Interestingly, a metabolic signature of glucose hypermetabolism and hypometabolism has been reported during ictal and inter-ictal periods of seizure activity, respectively (Theodore, 1999; Guo *et al.*, 2009; Kumar *et al.*, 2012; Shultz *et al.*, 2014; Patel, 2018). Although temporal hypometabolism has been reported in most patients with intractable temporal lobe epilepsy, the concept of glucose hypometabolism is more prevalent in chronic epilepsies (Guo *et al.*, 2009; Matheja *et al.*, 2001; Zilberter and Zilberter, 2017). Hypometabolism associated with seizures may reflect glucose transport abnormalities or defects in mitochondrial oxidative phosphorylation enzymes (Tenney *et al.*, 2014). Interestingly, clinical efficacy of ketogenic diets (KD) in Dravet syndrome patients suggests a critical role for energy metabolism, particularly its ability to enhance mitochondrial fatty acid utilization and glucose-sparing in genetic epilepsies. KD treatment is very effective as the diet provides alternative fuels for mitochondria and can reduce seizures by up to 90% in some patients with Dravet syndrome (Kinsman *et al.*, 1992; Kang *et al.*, 2005; Kossoff *et al.*, 2009; Nabbout *et al.*, 2011; Veggiotti *et al.*, 2011; Laux and Blackford, 2013; Gano *et al.*, 2014; Dressler *et al.*, 2015). Mitochondrial dysfunction can underlie hypometabolism concomitant with alterations in glucose metabolism, suggesting a co-ordinated response to an epilepsy-causing genetic mutation. Consistent with this, patients with Dravet syndrome muscle biopsies exhibit mitochondrial defects (Doccini *et al.*, 2015).

Using a larval zebrafish model of Dravet syndrome (*scn1lab*), we previously reported hypometabolism in the form of decreased glycolysis and mitochondrial oxygen consumption as a prominent characteristic of the mutants (Kumar *et al.*, 2016). The use of larval zebrafish has emerged as a promising whole-animal vertebrate model system due to its high degree of genetic homology to humans, small size, high reproducibility, rapid developmental rate and cost-effectiveness (Malicki, 2000; Chen and Ekker, 2004; Howe *et al.*, 2013). Additionally, their capacity to be used in multi-well assays with microliter amounts of media and rapid diffusion of media-dissolved drugs through the skin have made them quite popular for rapid high-throughput *in vivo* drug screening assays (Zon and Peterson, 2005; Rubinstein, 2006; Schlegel and Stainier, 2007; Hortopan *et al.*, 2010; Delvecchio *et al.*, 2011; Lessman, 2011; Rinkwitz *et al.*, 2011; Griffin *et al.*, 2017). The *scn1la*b mutant, a pre-clinical animal model originally identified in a chemical mutagenesis screen, recapitulates many aspects of the Dravet syndrome disease condition such as spontaneous unprovoked seizures, pharmaco-resistance, sleep disturbance, oculomotor deficit and early death (Schoonheim *et al.*, 2010; Baraban *et al.*, 2013; Hong *et al.*, 2016; Grone *et al.*, 2017). Demonstrating the true translational value of this model, two different serotonin receptor agonists (lorcaserin, Belviq^©^ and trazodone, Desyrel^©^) identified in *scn1lab* mutant seizure screens have shown clinical efficacy in small compassionate-use trials with an additional identified serotonin agonist (clemizole) currently in Phase II clinical trials and a serotonin re-uptake inhibitor (fenfluramine, Fintepla^©^) recently approved by the Federal Drug Administration for clinical treatment of Dravet syndrome (Dinday and Baraban, 2015; Sourbron *et al.*, 2016, 2017; Devinsky *et al.*, 2017, 2018; Griffin *et al.*, 2017; Kauppila *et al.*, 2018; Tolete *et al.*, 2018) (https://clinicaltrials.gov/ct2/show/NCT04462770?term=clemizole&draw=2&rank=3). Using a novel methodology to assess real-time bioenergetics, we previously reported significantly lower baseline glycolytic rates and mitochondrial respiration in *scn1lab* mutants compared to the wild-type controls (Kumar *et al.*, 2016). Remarkably, KD treatment effectively rescued altered metabolism to control levels resulting in attenuation of seizure activity in mutants (Baraban *et al.*, 2013; Kumar *et al.*, 2016). Thus, two lines of evidence validate the *scn1lab* mutant zebrafish model to study metabolic defects in human epilepsies. First, zebrafish and mammals regulate glucose similarly since the key gluco-regulatory mechanism is evolutionarily conserved among them (Elo *et al.*, 2007; Eames *et al.*, 2010). Second, glucose hypometabolism occurs in the brain of patients with Dravet syndrome with *SCN1A* mutations and zebrafish *scn1lab* mutants (Ferrie *et al.*, 1996; Korinthenberg *et al.*, 2004; Benedek *et al.*, 2006; Guerrini *et al.*, 2011; Sundaram *et al.*, 2013; Kumar *et al.*, 2016, 2018).

Interestingly, metabolic deficits in larval *scn1lab* mutants are accompanied by down-regulation of phosphoenolpyruvate carboxykinase (PEPCK)-encoded genes *pck1* and *pck2* (Kumar *et al.*, 2016). PEPCK, encoding gluconeogenic genes *pck1* (PEPCK-C, cytosolic) and *pck2* (PEPCK-M, mitochondrial), plays a vital role in metabolism as the enzyme has been almost exclusively linked to gluconeogenesis (Yang *et al.*, 2009). Deficiency in *pck* gene has been linked to a rare, autosomal recessive disorder associated with metabolic defects, muscle wasting and seizures, suggesting a critical role for *pck*/PEPCK in metabolic function and seizure control (Van Den Berghe, 1996). PEPCK is the primary enzyme that provides glucose during development and post-natal life (Jurczyk *et al.*, 2011). In larval zebrafish, the *pck* gene is used as a surrogate marker for glucose and a way to measure glucose in the whole organism (Elo *et al.*, 2007; Gut *et al.*, 2013). The rationale for our focus on PEPCK is 2-fold. First, it has been established that PEPCK is the primary source of glucose during development. Second, there are reports, suggesting that several drug classes (serotonergic compounds, corticosteroids, branched chain amino acids and KD) increase PEPCK flux via *pck* up-regulation (Elo *et al.*, 2007; Caraballo, 2011; Jurczyk *et al.*, 2011; Gut *et al.*, 2013; Kumar *et al.*, 2016). Both patients with Dravet syndrome and *scn1lab* mutant fish benefit from serotonergic compounds or KD, suggesting their metabolic actions may be mediated by a common pathway via PEPCK (Caraballo, 2011; Kumar *et al.*, 2016). Since both *scn1lab* mutants and patients with Dravet syndrome show hypometabolism, improvement of PEPCK activity or expression could ‘normalize’ metabolic deficits in *scn1lab* zebrafish to achieve the normal range of glycolysis and consequent substrate flux into mitochondrial energy production. We thus hypothesize that impaired glucose regulation may be a primary cause of metabolic defects and seizure behaviour in Dravet syndrome.

In this study, we asked if normalization of glucose levels via *pck* up-regulation in *scn1lab* mutant larvae improved phenotypic defects. We performed a metabolism-based small library screen with commercially available compounds that increased gluconeogenesis via up-regulation of *pck* gene expression (or PEPCK flux). The targeted screen identified compounds which up-regulate the expression of *pck1*, but not *pck2* more than 2-fold in *scn1lab* mutant larvae. The major finding in this study is that up-regulation of *pck1* by a translocator protein (TSPO) ligand PK11195 reversed metabolic deficits and electrographic seizures in the *scn1lab* mutants.

## Materials and methods

### Statement on the ethical treatment of animals

The study was carried out in strict accordance with the recommendations in the Guide for the Care and Use of Laboratory Animals of the National Institutes of Health (NIH). All procedures were approved by the Institute Animal Care and Use Committee (IACUC) of the University of Colorado Denver (UCD), which is fully accredited by the American Association for the Accreditation of Laboratory Animal Care. All experiments were designed to minimize pain and discomfort and conform to NIH regulatory standards of care.

### Zebrafish housing and husbandry

Zebrafish (*Danio rerio*) were maintained in the Animal Core Facility located at UCD according to standard procedures (Westerfield, 1993). Zebrafish were housed in a temperature-controlled facility under the standard light/dark (14:10 h) cycle. The temperature was tightly regulated and varied from 27 to 29°C (Westerfield, 1995). Water quality was monitored automatically and dosed to maintain conductivity (1000–1050 μs) and pH (6.95–7.30). The filtration system consisted of a bead filter, fluidized bed biofilter, finishing filters (bag filters) and UV sterilization. Zebrafish embryos were raised at 28.5°C in embryo medium consisting of 0.03% Instant Ocean (Aquarium Systems, Inc., Mentor, OH, USA) in deionized water containing 0.2 ppm methylene blue as a fungicide (Kumar *et al.*, 2016). Larvae were maintained in round Petri dishes (depth, 20 mm) in an incubator with a similar light–dark cycle. The housing density was limited to ∼50 larvae per dish. Adult zebrafish were housed in 1.5‐l tanks with stock density of 5–10 fish/tank and fed twice daily with adult commercial zebrafish diet (Gemma, 300).

### Zebrafish lines

The zebrafish strains used in this study were Tüpfel long fin and homozygous mutants *scn1lab*^*−*^^*/*^^*−*^ (*didy^s552^*) maintained on a Tüpfel long fin strain background (Baraban *et al.*, 2013). The *scn1lab*^*−*^^*/*^^*−*^ larvae were obtained by crossing adult *scn1lab^+/^*^*−*^ zebrafish and were sorted by their unique dark pigmentation. All experiments were performed with 5- to 6-days post-fertilization (dpf) larvae which were randomly selected as sex determination was not possible at the early stages (Griffin *et al.*, 2017). One limitation of the *scn1lab* mutant larvae that restricts long-term studies is that they have a shorter life span and they die prematurely between 10 and 12 dpf (Novak *et al.*, 2006; Baraban *et al.*, 2013).

### Drug dilutions

All drugs were purchased from Sigma-Aldrich and reconstituted in dimethylsulphoxide (DMSO) at a final concentration of 20 mM and stored at −20°C. On the day of assay, stock solutions were diluted with embryo media and pH adjusted to 7–7.5 to prepare the working concentration. Final DMSO concentration for all drug dilutions was ∼1%. All drug solutions were coded and a second investigator blinded to the drug identity performed the assays. The drug-treated larvae were monitored for good health and toxicity. Every larva was checked under the microscope to rule out the possibility of poor health based on two criteria; a visible beating heart and movement in response to touch or external stimulus. To ensure drug specificity, we ran drug-treated larvae with controls in the same plate with larvae from the same clutch. To address toxicity, a dose response for each compound was performed on wild-type fish before starting any experiment. A maximum tolerated dose was established for the drugs based on the above-mentioned criteria. Drugs were considered lethal or toxic if no visible heartbeat or movement in response to touch (external stimulus) was detected in at least 50% of the test fish after a 90-min exposure to drug (Baraban *et al.*, 2013; Griffin *et al.*, 2017).

### Gene expression studies

For qPCR, ∼10–12 *scn1lab* mutant larvae (6 dpf) treated with drugs (or DMSO as vehicle) were pooled and homogenized in TRIzol Reagent (Invitrogen). Total RNA was isolated using RNeasy spin columns (Qiagen) and quantified with a Nanodrop-1000 spectrophotometer (Thermo Scientific). The isolated RNA was used to synthesize cDNA using the High-Capacity cDNA Reverse Transcription Kit (Applied Biosystems). Universal PCR Master Mix and custom zebrafish-specific TaqMan probes-*acta1*, *pck1*, *pck2*, *gck* and *Tspo* (Applied Biosystem) were used for running the real-time PCR using a 7500 ABI Real-Time PCR System (Applied Biosystem). Relative gene expression was determined using the ΔΔCt comparative method to calculate fold change (RQ). The experimental unit for the qPCR experiments was as follows: For *pck1* and *pck2*, *n* = 4 (vehicle, PK11195, AC-5216, Ro5-4864 and stiripentol); *n* = 3 (remaining drugs), *gck* (*n* = 3) and *Tspo* (*n* = 3).

### Swim behaviour monitoring

For tracking zebrafish larvae swim behavior, we followed an acute locomotion assay protocol using DanioVision system running EthoVision XT software (Noldus Information Technology, Leesburg, VA, USA) as established previously (Baraban *et al.*, 2013; Dinday and Baraban, 2015; Griffin *et al.*, 2017, 2018, 2020). Briefly, 5–6 dpf *scn1lab* mutant larvae were placed individually in 96-well plates with embryo media in each well. The larvae were first acclimatized by placing them in the DanioVision chamber under dark light for a 20-min period followed by a 10-min baseline recording epoch. After obtaining baseline measurements, the embryo media was replaced with 75-μl test drugs (at specific concentrations) or 1% DMSO (vehicle) in control wells and incubated for a period of 3-h. The treatment period was followed by a 10-min experimental recording epoch. Criteria for a positive hit with the threshold set at ≥40% mean velocity based on the previous standard deviations was followed as established by Baraban *et al.* (2005). The experimental unit for this assay was *n* = 36 of *scn1lab* mutants for each drug treatment. Two independent locomotion screens (screen1 and screen 2) were performed in two facilities; University of Colorado, Denver (UCD) and University of California, San Francisco (UCSF), respectively. Coded drugs were used for screening and the analysis was performed by investigators blinded to the compounds. The 3-mercaptopicolinic acid (3-MPA) studies were performed at UCD with *n* = 24 for the wild-type control group and drug-treated group.

### Metabolic measurements

Baseline glycolysis and mitochondrial respiration rates (ECAR, Extracellular Acidification Rate and OCR, Oxygen Consumption Rate, respectively) were simultaneously measured in live 6 dpf *scn1lab* mutant larvae with Seahorse XF24 Extracellular Flux Analyzer (Agilent) as described previously (Stackley *et al.*, 2011; Kumar *et al.*, 2016). In brief, 6-dpf *scn1lab* larvae were exposed to drug treatment (10 μM) or control DMSO (1%) using a 3-h exposure protocol. Drug/DMSO-treated larvae were individually loaded per well of a 24-well islet plate with a mesh screen placed on top to hold the fish in place as described in Kumar *et al.* (2016) and Stackley *et al.* (2011). For testing mitochondrial function, the XF Cell Mito Stress Test (Seahorse Bioscience, Agilent) was used as per manufacture’s guidance. Three inhibitors, oligomycin, trifluoromethoxy carbonylcyanide phenylhydrazone (FCCP) and Sodium Azide, were used which target the functions of ATP synthase, mitochondrial uncoupling and complex I/III, respectively. All drugs were prepared by following the manufacturer’s recommendations and were dissolved in embryo water. The inhibitors were loaded into XF Extracellular Flux assay plates (Agilent). Titrations were performed to determine the optimal concentration of each inhibitor. Proton leak, ATP-linked respiration and spare respiratory capacity were calculated by the XF Cell Mito Stress Test Report Generator (Agilent). The experimental unit for these assays are *n* = 10 for both baseline ECAR and OCR; *n* = 20 for XF Cell Mito Stress Test.

### Glucose measurements

For measuring absolute glucose levels, lysates were prepared by pooling exactly 20 zebrafish larvae with/without drug treatment and briefly sonicated (on ice) in 160 μl ice-cold phosphate-buffered saline. The volume of phosphate-buffered saline was equivalent to 8μl per larva. Samples were centrifuged for 10 min at 12 000 rpm and the glucose was quantified using an Amplex Red Glucose/Glucose Oxidase Assay kit (Invitrogen). The reaction was assembled and glucose standard curves were generated for every plate as per the manufacturer’s guidance. For our experiment, 50 μl of sample buffer composed of 8 μl of sample lysate and 42 μl of assay buffer (supplied with the kit) was used, and fluorescence (excitation 535 nm; emission, 590 nm) was detected using a plate reader (BioTek Synergy 4). As per manufacturer’s instructions, the fluorescence values were corrected by subtracting measurements from control reactions without sample. Standard curves were generated and the absolute glucose values were interpolated. The absolute glucose values were normalized with the protein content which was measured by a standard Bradford assay using Coomassie Protein Assay Kit (Pierce). For drug treatment assay *n* = 11 for vehicle and PK11195; *n* = 6 for rest of the drugs. For 3-MPA studies, *n* = 5 for the wild-type control group and drug-treated group. For all experiments, samples were measured in triplicates and each experiment repeated a minimum of three times.

### Electrophysiology

Freely behaving 6-dpf *scn1lab* larvae were exposed to PK11195 drug treatment (10 μM) or control embryo media using a 3-h exposure protocol. The coded larvae were randomly selected by a second investigator blinded to the drug exposure group, paralysed with pancuronium (300 μM) and immobilized in 2% low-melting agarose. Local field potential (LFP) recordings were obtained from forebrain structures using a single-electrode recording at room temperature as described previously (Baraban *et al.*, 2005, 2013; Griffin *et al.*, 2017, 2020). Briefly, a single LFP glass microelectrode was placed, under visual guidance, in optic tectum and 10-min recording epochs were obtained using Axoclamp software (Molecular Devices; Sunnyvale, CA) at an acquisition rate of 1 kHz. Electrophysiological recordings were analysed *post hoc* using Clampfit software (Molecular Devices). Burst frequency was determined by counting the number of epileptiform events per minute during a 10-min recording epoch. Burst duration was determined by measuring the onset-to-offset interval for all events during the same epoch. At the end of the recordings, larvae were gently freed from agarose and returned to a Petri dish containing embryo media. All experiments were performed in the UCSF facility on at least three independent clutches of *scn1lab* zebrafish larvae (*n* = 16 for both control- and drug-treated groups).

### Statistical analyses

Statistical analyses were performed with Graphpad Prism 7.0 for Windows. All data were represented as mean ± SEM unless stated otherwise. Statistical analyses included Student’s two-tailed unpaired *t*-test followed by Welch’s correction and ANOVA followed by Dunnett’s Multiple comparison tests. For all experiments, significance was taken as *P <* *0.05, **P <* *0.01, ***P <* *0.001 and ****P <* *0.0001 unless otherwise stated.

### Data availability

The data that support the findings of this study are available from the corresponding author, upon reasonable request.

## Results

### The *scn1lab* mutants have impaired mitochondrial bioenergetics and are hypoglycaemic

**Figure 1:**
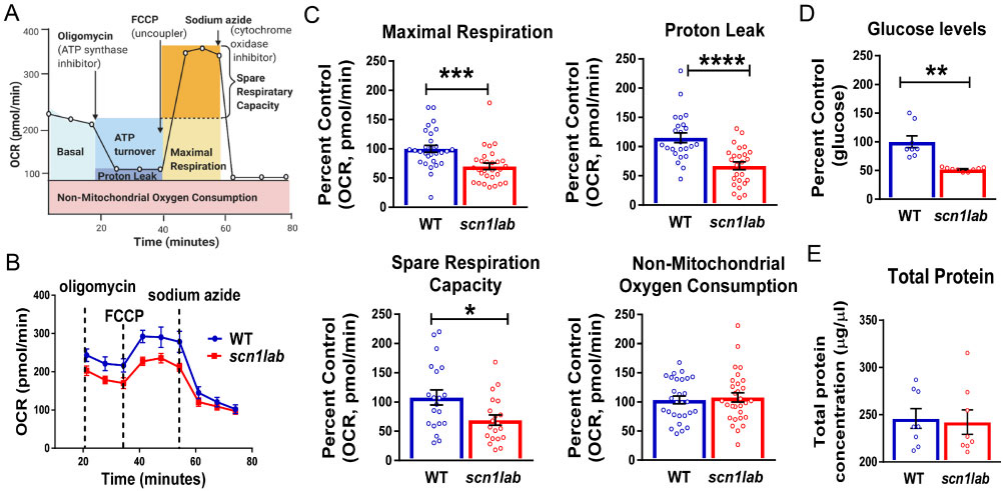
**The *scn1lab* mutants have impaired mitochondrial bioenergetics and are hypoglycemic.** (**A**) A schematic representation of a complete metabolic profile of mitochondrial respiration for measuring different mitochondrial indices using the XF Seahorse Mito Stress Test. Agilent Technologies, Inc.^©^ 2020, adapted and reproduced with permission, courtesy of Agilent Technologies, Inc. Figure created with BioRender.com. (**B**) The Seahorse Bioanalyzer was used to measure OCR in 6-dpf *scn1lab* and wild-type larvae by sequentially adding oligomycin, FCCP and sodium azide (instead of antimycin A/rotenone). A representative OCR profile plot from a single run represents decreased OCR in *scn1lab* mutant compared to wild-type group. (**C**) Bar graphs represent mitochondrial metabolic parameters. There is a significant decrease in maximal respiration, proton leak and spare respiratory capacity in *scn1lab* mutants compared to the wild-type larvae. There is no significant difference in non-mitochondrial consumption rate. Data are presented as mean ± SEM and are normalized to the control group (wild type). Statistics performed by unpaired-*t* test with Welch’s correction. Asterisks (*) indicate a significance of difference between the two groups at *P <* *0.05, **P <* *0.01 and ***P <* *0.001 and ****P <* *0.0001; the data points represent each larva, *n* = 20 for each group. (**D**) Representative bar graph depicting glucose levels in 6-dpf *scn1lab* and wild-type larvae. There is a significant decrease in glucose levels in the *scn1lab* mutants compared to the wild-type larvae. Data are presented as mean ± SEM and are normalized to the control group (wild type). (**E**) Representative bar graph depicting total protein levels in 6-dpf *scn1lab* and wild-type larvae. No significant difference is observed in the total protein levels between the two groups. Data are presented as mean ± SEM. For (D) and (E), statistics were performed by unpaired-*t* test with Welch’s correction. Asterisks (*) indicate a significance of difference between the two groups at **P <* *0.01. *n* = 8, where each sample represents 20 pooled larva. In all panels, WT refers to wild-type larvae.

We previously reported metabolic deficits in s*cn1lab* and *stxbp1b* mutant zebrafish lines (Grone *et al.*, 2016; Kumar *et al.*, 2016). Both basal ECAR (a measure of glycolytic rate) and OCR (a measure of mitochondrial respiration) were significantly lower in mutants. To validate and further characterize oxidative metabolic defects in *scn1lab* mutants, we performed a Mitochondrial Stress Test on 6-dpf wild-type (*n* = 20) and *scn1lab* mutant larvae (*n* = 20) using the Seahorse XF Flux Analyzer (Stackley *et al.*, 2011; Gibert *et al.*, 2013; Kumar *et al.*, 2016; Ibhazehiebo *et al.*, 2018). The stress test is designed to test bioenergetic function of mitochondria by measuring various key parameters such as basal respiration, maximal respiration, proton leak, spare respiratory capacity and non-mitochondrial respiration after injections of inhibitors and an uncoupler. As shown in the schematic representation of the assay (Fig. 1A), these injections are typically oligomycin—an inhibitor of ATP synthase (complex V), followed by FCCP—an uncoupling agent and finally a combination of rotenone/antimycin A—complex I and III inhibitors, respectively. Maximal respiration and proton leak are calculated after the addition of FCCP and oligomycin, respectively. Spare respiratory capacity is calculated based on the difference between the basal respiration and the maximal respiration. Figure 1B represents an individual run with the pharmacological inhibitors to measure the bioenergetic profile of *scn1lab* mutants and wild-type control larvae. Here, we used sodium azide; an inhibitor of cytochrome *c* oxidase (complex IV) and the ATP synthase (complex V) instead of antimycin A/rotenone as it proved to be a better non-toxic choice for the larvae. Figure 1B clearly shows that the OCR is lower in mutants compared to the wild-type controls. Interestingly, we observed deficits in three key parameters of mitochondrial function in *scn1lab* mutants: (i) maximal respiration, (ii) spare respiratory capacity and (iii) proton leak (Fig. 1C).

To further investigate hypometabolic characteristics of larval *scn1lab* mutants, we measured absolute glucose levels. Since *pck* levels are important for larval glucose regulation (Elo *et al.*, 2007), we assayed glucose levels in whole *scn1lab* mutants (*n* = 8) and wild-type sibling controls (*n* = 8). We observed significantly lower glucose levels in *scn1lab* mutants compared to wild type (Fig. 1D). No significant differences were observed in total protein concentrations between *scn1lab* mutants (*n* = 8) and wild-type (*n* = 8) (Fig. 1D). Taken together, our results indicate that *scn1lab* mutants have defective mitochondrial respiration and are hypoglycaemic, suggesting an overall decrease in both major energy-producing pathways.

### Reduction in convulsive seizure-like swim behaviour with validated *pck1* activators

**Figure 2:**
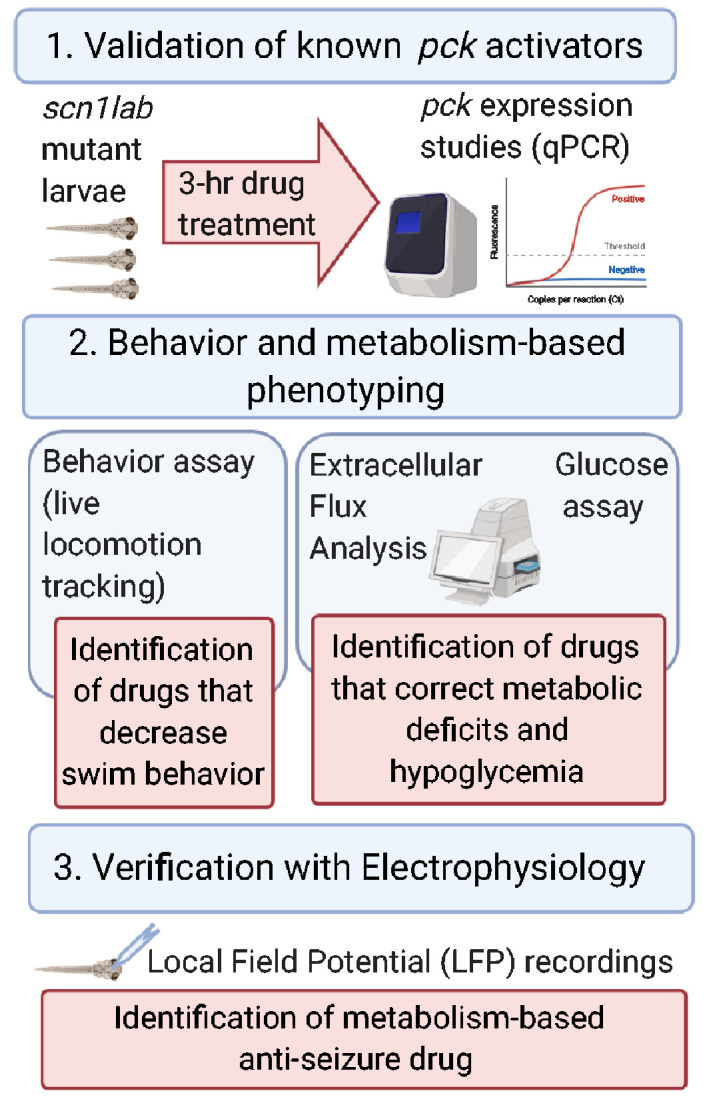
**Schematic representation of the workflow of the experimental strategy.** The three main steps to identify metabolism-based anti-epileptic drugs for Dravet syndrome include: validation of *pck* activators, behaviour and metabolism-based phenotyping and finally verification with electrophysiology. (Figure created with BioRender.com).

Metabolic defects in *scn1lab* zebrafish larvae are accompanied by decreased expression levels of key gluconeogenic genes *pck1* and *pck2* which encode PEPCK-C and PEPCK-M in the cytosol and mitochondria, respectively (Kumar *et al.*, 2016). Larval zebrafish share close similarities with mammals in regulating glucose metabolism and PEPCK has been identified as a biomarker of glucose levels. The strategy to up-regulate gluconeogenesis by increased expression of *pck1/2* or increased PEPCK flux provides an alternate pathway (i.e. independent of glycolysis) to improve metabolism. Previous reports suggest that several drug classes such as serotonin, steroids, branched chain amino acids and KD increase PEPCK flux via *pck* up-regulation (Caraballo, 2011; Gut *et al.*, 2013; Kumar *et al.*, 2016). To determine if pharmacological manipulation of *pck1/2* is therapeutic in *scn1lab* mutants, we first selected a small group of commercially available known *pck*/PEPCK flux activators from various drug classes (Table 1). A schematic representation of the workflow is shown in Fig. 2, which outlines three main steps. In the first step, the ability of *pck* activators to increase *pck* expression was evaluated in larval zebrafish model, and only those capable of inducing a 2-fold increase in *pck* expression (*pck1* and/or *pck2*) progressed. In the second step, the validated *pck* activators were screened in two platforms; behaviour and metabolism, independently. Only drugs that were positive hits in both behaviour and metabolic platforms were taken to the third step, in which electrophysiology was examined.

**Table 1:** Screen composed of *pck* activators tested in *scn1lab* mutants

Drugs	Class	*pck1* Expression	Behavior		Metabolism		
			Screen 1	Screen 2	ECAR	OCR	Glucose
PK11195^a^	TSPO ligands (antagonist, agonist)	+	+	+	+	+	+
AC-5216^b^		+	+	ND	−	−	−
Ro5-4864^c^		+	−	−	−	−	−
9-*cis* Retinoic acid	Vitamin A	+	−	−	+	+	+
Fluoxetine^b^	SSRI	+	+	+	−	−	−
Clofibrate^d^	Fatty acids and fibrates	NC	−	−	−	−	−
Oleate		+	−	−	−	−	−
8-Bromo cAMP	cAMP analog	+	−	−	−	−	+
Formoterol	Beta-adrenergic Agonists	+	−	−	−	+	+
Isoprenaline		+	−	−	−	+	+
Ractopamine		+	−	−	−	−	+
Oxaloacetate	PEPCK flux substrate	NC	−	−	−	−	−
Stiripentol^e^	Anti-convulsant	NC	+	+	−	−	−

+ Positive for the assay; − Negative for the assay; ND, not determined; NC, no change;

a Drug positive for all assays;

b Positive for screen 1 of behaviour assay but negative for all metabolic assays;

c Hyper-excitability in both screens of behaviour assays;

d No change in *pck1* expression, but tested as a negative control for all assays,

e Positive control for all assays.

**Figure 3:**
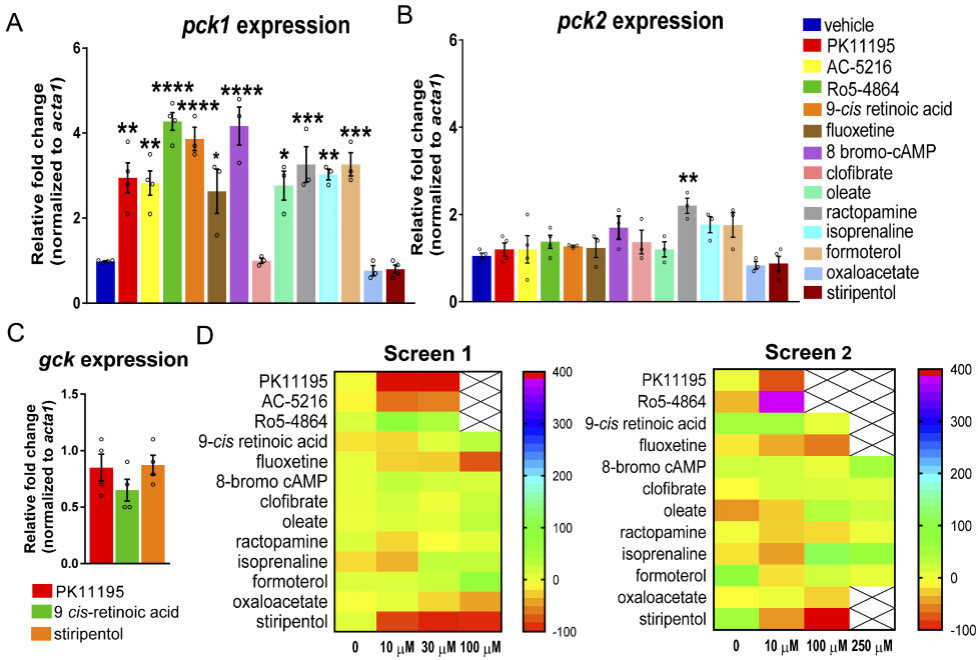
**Reduction in convulsive seizure-like swim behaviour with validated *pck1* activators.** (**A**) The bar graph represents qPCR data with relative fold change of *pck1* gene in 3-h drug-treated *scn1lab* mutants. The drugs that had more than 4-fold up-regulation of *pck1* were 9-*cis* retinoic acid, Ro5-4864 and 8-bromo-cAmp and with more than 2-fold up-regulation were PK11195, AC-5216, fluoxetine, isoprenaline, formoterol, oleate and ractopamine. (**B**) The bar graph represents qPCR data with relative fold change of *pck2* gene in 3-h drug-treated *scn1lab* mutants. No significant up-regulation of *pck2* was observed except with ractopamine. (**C**) No change in expression of *gck* gene was observed in drug-treated *scn1lab* mutants. Drugs include PK 11195, 9-*cis* retinoic acid and stiripentol. Data for **A**, **B** and **C** were normalized to the housekeeping gene *acta1* and presented as mean ± S.E.M. Statistics were performed by one-way ANOVA followed by Dunnett’s multiple comparison test with significance taken as *P <* *0.05, **P <* *0.01, ***P <* *0.001 and ****P <* *0.0001. Values represent averages from *n* = 4, where each sample represents 10–12 pooled 6-dpf *scn1lab* mutant larvae. (**D**) Behaviour-based screening of validated *pck1* activators. Heat maps representing change in mean velocity of *scn1lab* mutants in response to the 13 compounds with 3-h treatment. The heat maps represent two independent screens of behaviour phenotyping in *scn1lab* mutants. Screen 1 represents percent change in mean velocity (mm s^−1^) in *scn1lab* larvae treated with three concentrations (10, 30 and 100 μM) of the drugs for 3-h (performed in UCD facility). Screen 2 represents behaviour screen performed in UCSF facility with three concentrations (10, 100 and 250 μM). The locomotion assay was recorded for 10 min in Danio Vision system using locomotion tracking software (EthoVision XT 11.5-Noldus Information Technology). PK11195 at 10 and 30 μM; AC-5216 at 10 and 30 μM; fluoxetine at 100 μM shows significant decrease in mean velocity (threshold set as ≥40%). Stiripentol was used as a positive control in both screens. Each box represents the percent change in velocity from independent locomotion assays with at least six *scn1lab* larvae (*n* = 36). Toxic dose is represented with a cross and the scale at the right-hand side represents the range in percent change in mean velocity.

To validate up-regulation of *pck* expression, we treated 6-dpf *scn1lab* mutant larvae with the selected compounds (10 μM) for 3-h and performed qPCR to measure the expression levels of *pck1* and *pck2* (*n* = 4, vehicle, PK11195, AC-5216, Ro5-4864 and stiripentol; *n* = 3, remaining drugs). Interestingly, we found more than a 2-fold increase in *pck1* expression for the majority of compounds: PK11195, AC-5216, fluoxetine, isoprenaline, oleate, formoterol and ractopamine (Fig. 3A). Additionally, we found a more than 4-fold increase in *pck1* expression for Ro5-4864, 9-*cis* retinoic acid and 8-bromo-cAmp (Fig. 3A). We did not observe an increase in *pck2* expression for most drugs with the exception of ractopamine, which showed more than 2-fold increase (Fig. 3B). Predictably, oxaloacetate, which is known to increase flux through PEPCK and stiripentol, an antiepileptic drug used in Dravet syndrome patients did not up-regulate either *pck1* or *pck2* (Fig. 3A and B). Clofibrate did not increase either *pck1* or *pck2* expression (Fig. 3A and B). The expression studies indicate that pharmacologically targeting *pcks* results in significant up-regulation of particularly *pck1* in *scn1lab* mutant larvae. Furthermore, 3-h incubation with *pck* activators PK11195 and 9-*cis* retinoic acid, (*n* = 4) increased *pck1* expression but not the glycolytic gene glucokinase (*gck*), thus validating specificity of the expression data (Fig. 3C). Moreover, the antiepileptic drug stiripentol did not alter *pck1*, *pck2* or *gck* expression (*n* = 4). This finding indicates that the anti-epileptic property of stiripentol is not dependent on gluconeogenesis and it functions via its known allosteric actions on GABA-A receptors (Fisher, 2011).

To test if *pck1* activators validated from the expression studies could pharmacologically restore behavioural phenotypes, we tracked swim activity. Prior to tracking, 5–6 dpf *scn1lab* mutant larvae were first treated for 3-h with *pck1* activators at three different concentrations 10, 30 and 100 μM dissolved in embryo media (*n* = 36 per group). Next, a second investigator blinded to drug exposure monitored convulsive seizure-like swim behaviours using a DanioVision system with automated locomotion tracking software (EthoVision XT 11.5, Noldus Information Technology). The threshold for a significant effect on locomotion was set at −40% (Baraban *et al.*, 2013; Griffin *et al.*, 2017, 2018, 2020). This value is based on a large database composed of control trials in untreated *scn1lab* mutants (vehicle). Among 13 tested compounds, we identified three compounds PK11195 (10 and 30 μM), AC-5216 (10 and 30 μM) and fluoxetine (100 μM) that significantly decreased swim behaviour in *scn1lab* mutants (screen 1, Fig. 3D). Additionally, PK11195 (10 μM) and fluoxetine (100 μM) were validated in an independent DanioVision-based behavioural screen in the UCSF lab (screen 2, Fig. 3D). Stiripentol was used as a positive control in both screens (Baraban *et al.*, 2013). Taken together, we identified the top ranking drug PK11195, that up-regulated *pck1* expression and reduced convulsive seizure-like swim behaviours in *scn1lab* mutants from two independent screens.

### Correcting metabolic deficits and hypoglycaemia with validated *pck1* activators

**Figure 4:**
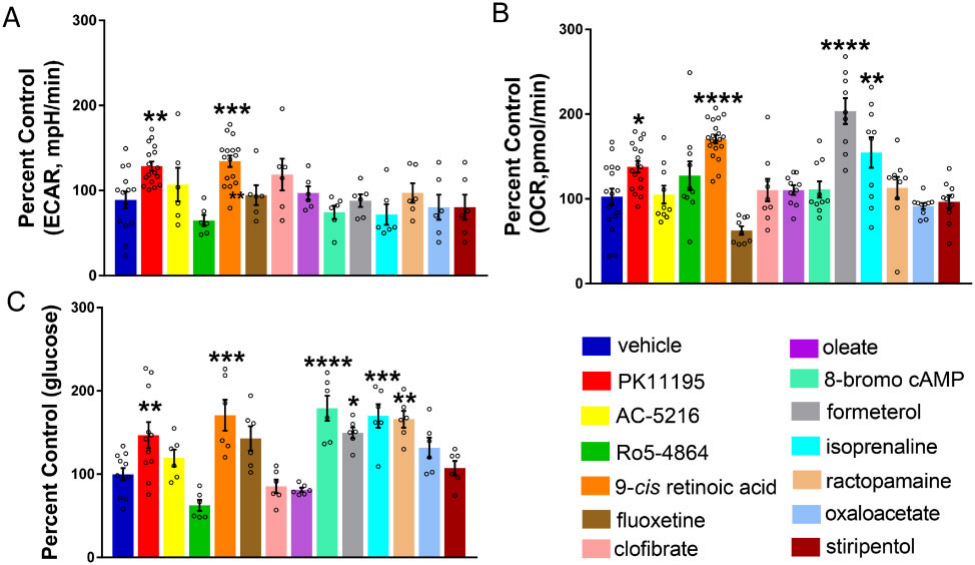
**Correcting metabolic deficits and hypoglycaemia with validated *pck1* activators.** (**A**) Representative bar graph showing baseline ECAR in 6-dpf *scn1lab* mutants treated with *pck1* activators. A significant increase was observed in ECAR for PK11195 and 9-*cis* retinoic acid. Data are presented as mean ± SEM normalized to wild-type controls. Statistics were performed by one-way ANOVA followed by Dunnett’s multiple comparison test with significance taken as **P <* *0.01 ***P <* *0.001 and ****P <* *0.0001 (*n* = 10). (**B**) Representative bar graph showing baseline OCR in *scn1lab* mutants treated with *pck1* activators. A significant increase in OCR was observed for PK11195, 9-*cis* retinoic acid, formoterol and isoprenaline. Data are presented as mean ± SEM normalized to wild-type controls. Statistics were performed by one-way ANOVA followed by Dunnett’s multiple comparison test with significance taken as **P <* *0.01, ***P <* *0.001 and ****P <* *0.0001 (*n* = 10). (**C**) Representative bar graph showing glucose levels (normalized to total protein) in drug-treated *scn1lab* mutants. There is a significant increase in glucose levels in the *scn1lab* mutants treated with PK11195, 9-*cis* retinoic acid, formoterol, 8-bromo-cAMP and ractopamine, compared to the DMSO control (vehicle). Data are presented as mean ± SEM normalized to wild-type controls. Statistics were performed by one-way ANOVA followed by Dunnett’s multiple comparison test with significance taken as *P <* *0.05, **P <* *0.01 and ***P <* *0.001. Values represent averages from *n* = 11, vehicle, PK11195; (*n* = 6), other drugs, where each sample represents 10–20 pooled larvae. In all panels, WT refers to wild-type larvae.

Extracellular Flux Analyzers have a unique ability to measure *in vivo* metabolic phenotypes by simultaneously measuring respiration and glycolysis in real time. We previously reported metabolic deficits in s*cn1lab* and *stxbp1b* mutant zebrafish lines (Grone *et al.*, 2016; Kumar *et al.*, 2016). Mutations in the synaptic machinery gene syntaxin-binding protein 1 (*STXBP1*) are also linked to neurodevelopmental disorders (Saitsu *et al.*, 2008; Otsuka *et al.*, 2010; Carvill *et al.*, 2014; Tso *et al.*, 2014). To determine if the *pck1* activators would improve these metabolic deficits, we treated 6 dpf *scn1lab* mutants with 10 μM of the drugs for 3-h (*n* = 10). We simultaneously measured baseline glycolysis and mitochondrial respiration rates in *scn1lab* mutants with drug treatment or vehicle using the XF24 Flux Analyzer. Interestingly, PK11195 and 9-*cis* retinoic acid treatment normalized baseline glycolytic rates (ECAR) and mitochondrial respiration rates (OCR) (Fig. 4A and B), whereas formoterol and isoprenaline rescued only the levels of OCR (Fig. 4B).

Next, to determine if the *pck1* activators would improve hypoglycaemia in *scn1lab* mutants, we treated 6-dpf mutants with 10 μM of the drugs for 3-h (*n* = 11, vehicle, PK11195; *n* = 6, other drugs). Using the glucose oxidase-based fluorescence assay, we measured glucose levels in the drug-treated samples and compared them with vehicle control. Any significant improvement of glucose was considered as a normalization or correction of glycolytic levels in the drug-treated *scn1lab* mutants. Interestingly, PK11195, 9-*cis* retinoic acid, isoprenaline, ractopamine, 8-bromo cAMP and formoterol treatment significantly increased larval glucose levels (Fig. 4C). Thus, our results from the bioenergetic and glucose assays indicate that PK11195 corrects metabolic deficits (i.e. glycolytic rates, mitochondrial respiration and glucose levels) in *scn1lab* mutants.

### TSPO ligand, PK11195 is a promising therapeutic drug

**Figure 5:**
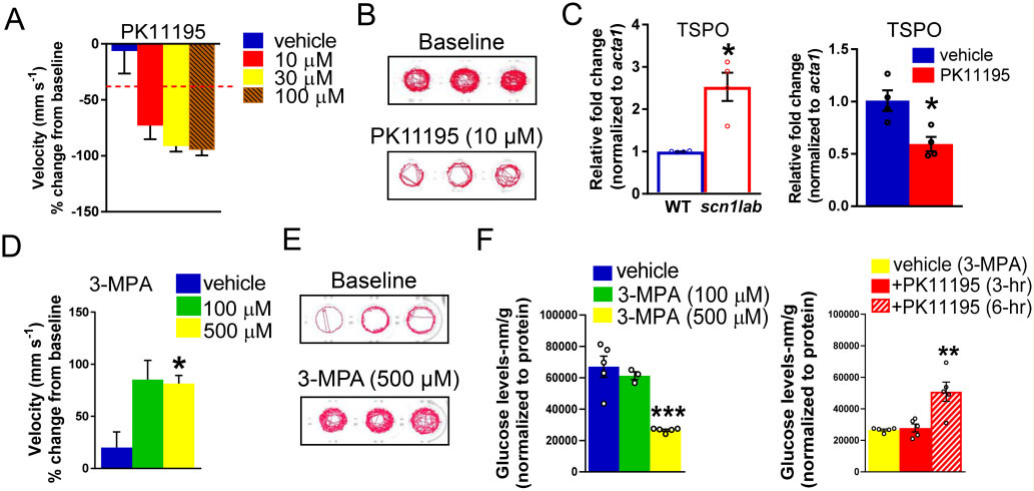
**PK11195 is a positive hit in both metabolic and behaviour phenotyping assays.** (**A**) Bar graph representing percent change in mean velocity (mm s^−1^) in *scn1lab* mutant larvae (6 dpf) treated with three concentrations of PK11195 (10, 30 and 100 μM) for 3-h. Locomotion was recorded for 10-min in DanioVision system using a locomotion tracking software (EthoVision XT 11.5-Noldus Information Technology). PK11195 at both 10 and 30 μM showed significant decrease in mean velocity. PK11195 at 100 μM was a toxic dose. Data are presented as mean change in velocity ± SEM with *n* = 36. The cut-off for significant decrease in mean velocity is set at ≥40% (dashed line). (**B**) Representative traces of locomotion plots obtained from 10-min recordings for baseline and 10 μM PK11195-treated larvae are shown. (**C**) The bar graph represents qPCR data with increased expression of *Tspo* mRNA in *scn1lab* mutants compared to wild-type. The bar graph on the right represents qPCR data with relative fold change of *Tspo* with 3-h PK11195-treated *scn1lab* mutants. No significant change was observed; however, a trend of decreased *Tspo* expression was seen in PK11195-treated *scn1lab* mutants. Data were normalized to the housekeeping gene *acta1* and presented as mean ± S.E.M. Statistics were performed by Student’s unpaired *t*-test. Asterisks (*) indicate a significance *P < 0.05. Values represent averages from *n* = 3, where each sample represents 10–12 pooled 6-dpf larvae. (**D**) The bar graph represents increased swim behaviour in 5-dpf wild-type larvae treated with 500 μM 3-MPA. No increase in swim behaviour was observed with 100 μM 3-MPA (*n* = 24). WT refers to wild-type larvae. (**E**) Representative traces of locomotion plots obtained from 10-min recordings for baseline and 500 µM 3-MPA-treated wild-type larvae are shown. (**F**) Representative bar graph showing glucose levels (normalized to total protein) in 3-MPA-treated wild-type larvae. A significant decrease of glucose levels was observed in 3-MPA-treated larvae (3- and 6-h) when compared with vehicle (wild-type untreated larvae). The bar graph in the right-hand side represents glucose levels in PK11195-treated larvae. A significant increase of glucose levels was observed in larvae that were incubated with 3-MPA (500 μM) and PK11195 (10 μM) for 6-h but not for 3-h compared with vehicle (3-MPA-treated larvae). Data are presented as mean ± SEM. Statistics were performed by two-way ANOVA followed by Tukey’s multiple comparison test with significance taken as **P <* *0.01, ***P <* *0.001 and ****P <* *0.0001. Values represent averages for *n* = 5, where each sample represents 20 pooled larvae.

Our metabolism-based small library screen with commercially available compounds identified drugs that increased gluconeogenesis via up-regulation of *pck1* gene expression (or PEPCK flux) in *scn1lab* mutants. The TSPO ligand, PK11195 was identified as our top candidate as it was the only drug that was a positive hit in all assays (Table 1). Most notably, PK11195 treatment increased *pck1* expression, normalized glucose levels, corrected metabolic deficits and significantly decreased seizure-like swim behaviour in mutant larvae. PK11195 is a well-known synthetic ligand of the 18-kDa TSPO, localized at the mitochondrial outer membrane. A 3-h treatment of PK11195 at 10 µM (*n* = 4) increased *pck1* expression more than 2-fold but not *pck2* expression. Our two independent locomotion screens identified PK11195 because its 3-h treatment could reduce convulsive seizure-like swim behaviours in *scn1lab* mutants (Fig. 5A and B, *n* = 36). Additionally, *scn1lab* mutants treated with PK11195 could significantly rescue both ECAR (*n* = 20) and OCR (*n* = 16) levels as well as glucose levels (*n* = 11) to wild-type controls. Given the higher expression of *Tspo* mRNA in various diseased models, we performed *Tspo* mRNA expression studies with *scn1lab* mutants and wild-type larvae. Interestingly, we observed more than 2-fold increase in *Tspo* expression in the mutants (Fig. 5C, *n* = 3). Since elevated TSPO expression has been linked with and used to detect neuroinflammation, we asked if PK11195 treatment altered *Tspo* expression in *scn1lab* mutants. Although we did not observe a statistically significant change in the expression levels but we did observe a trend of decreased *Tspo* mRNA after PK11195 treatment which suggests a possibility of the drug inhibiting the expression *Tspo* (Fig. 5C, *n* = 3).

To begin an investigation of the underlying mechanism of PK11195 and to assess if its action on gluconeogenesis is sufficient to rescue the defects in *scn1lab* mutants, we used an approach to inhibit the metabolic pathway. Because PEPCK enzymes are crucial for providing glucose in larval zebrafish during embryogenesis, the genetic deletion of *pck1* would be deleterious and thus not a feasible model. We therefore used a pharmacological approach to determine if transient inhibition of PEPCK-C in wild-type larvae would mimic the behaviour and metabolic phenotype observed in *scn1lab* mutants using a well-known *pck1* inhibitor, 3-MPA (Makinen and Nowak, 1983; Jurczyk *et al.*, 2011; Balan *et al.*, 2015). We monitored spontaneous swim behaviour in wild-type larvae treated with 3-MPA (*n* = 24) using the Danio Vision system with automated locomotion tracking software (EthoVision XT 11.5-Noldus Information Technology). Our results show that 3-MPA increased seizure-like swim behaviour in wild-type larvae (Fig. 5D and E). In Fig. 5E, the representative locomotion plots of 3-MPA-treated wild-type larvae indicate hyperactivity and convulsive seizure-like behaviour as seen in *scn1lab* mutants (Fig. 5B, see baseline locomotion plots). This kind of characteristic behaviour phenotype in *scn1lab* mutants has already been validated in the study of Baraban *et al.* (2013). Our data suggest that the 3-MPA-treated wild-type larvae mimics the behaviour phenotype of *scn1lab* mutants. Treatment of 5-dpf wild-type larvae for 3-h with 3-MPA (*n* = 5) decreased glucose levels in a dose-dependent manner, confirming its ability to inhibit PEPCK (Fig. 5F). Finally, we asked if PK11195 would normalize glucose levels in 3-MPA-treated wild-type larvae. Briefly, 5-dpf wild-type larvae were treated with 3-MPA (500 µM) and PK11195 (10 µM) and harvested at 3- or 6-h time point (*n* = 5). Incubation of wild-type larvae for 6-h, but not 3-h with PK11195, restored glucose levels to wild-type levels (Fig. 5F). These data further confirm the ability of PK11195 to normalize dys-regulated glucose metabolism.

### PK11195 treatment suppresses electrographic seizures

**Figure 6:**
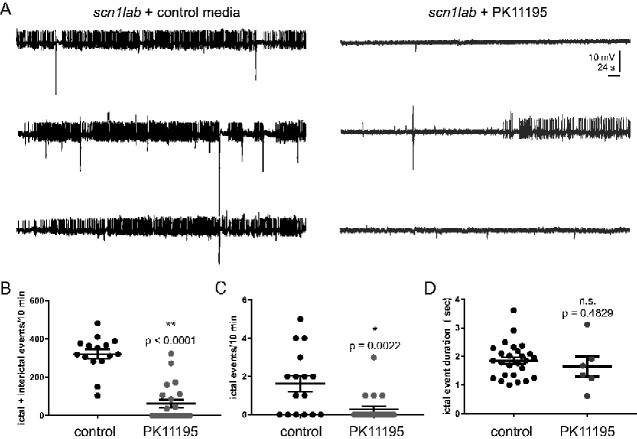
**PK11195 treatment effectively suppresses electrographic seizure activity.** (**A**) Representative 10-min local field LFP recording epochs with 6-dpf *scn1lab* mutants treated with control media and 10 μM PK11195 for 3-h. (**B**) The graph shows a significant reduction in the frequency of ictal- and inter-ictal-like electrographic seizure events with a 3-h 10 μM PK11195 exposure. (**C**) The graph shows a significant effect of 3-h 10 μM PK11195 exposure in significantly suppressing ictal events alone. (**D**) No change in ictal event duration was observed among the two groups. All drug-treated *scn1lab* mutant larvae were freed from agar post-experiment and the survival rate at 24-h was 94%. Data represent 6 dpf from three independent clutches, *n* = 17 for controls; *n* = 22 for PK11195 treatment. Data are presented as mean ± SEM; Student’s unpaired *t*-test was used. Asterisks (*) indicate a significance *P < 0.05; **P < 0.001.

Successful phenotype-based screening for anti-seizure activity in *scn1lab* mutant zebrafish larvae requires a secondary electrophysiology assay to discriminate compounds that effectively reduce, or suppress, electrographic seizure events from false-positives that only suppress behaviours (Baraban *et al.*, 2013; Dinday and Baraban, 2015; Griffin *et al.*, 2017, 2018, 2020). Prolonged 3-h exposure to PK11195 increased *pck1* expression (Fig. 3A) and reduced convulsive seizure-like swim behaviours observed in *scn1lab* mutants at 5 dpf in two independent screening assays (Figs. 3D and 5A and B). To confirm an action on electrographic seizure activity in the central nervous system, we first exposed freely behaving *scn1lab* larvae to 10 μM PK11195 (*n* = 22) or control embryo media (*n* = 21) using the same 3-h exposure protocol. Next, coded larvae were randomly selected by a second investigator blinded to drug exposure protocol, briefly paralysed with pancuronium, and immobilized in agarose for LFP recordings. A single LFP glass microelectrode was placed, under visual guidance, in optic tectum and 10-min recording epochs were obtained (Fig. 6A). Long-duration large-amplitude ictal-like and brief small-amplitude inter-ictal-like events were analysed offline using an event detection setting. We noted a significant reduction in the frequency of ictal- and inter-ictal-like electrographic seizure events with a 3-h 10 μM PK11195 exposure (Fig. 6B and C). No change in ictal event duration was noted (Fig. 6D). At the conclusion of some recordings, larvae were gently freed from agarose and returned to a Petri dish containing embryo media (*n* = 16); 94% of *scn1lab* larvae exposed to this protocol were alive and freely swimming at 24-h.

## Discussion

This study builds on our previous work that demonstrated metabolic deficits and down-regulation of key gluconeogenic genes, *pck1* and *pck2* in *scn1lab* mutants, a translatable zebrafish model of Dravet syndrome (Kumar *et al.*, 2016). Here, we further characterized the hypometabolic characteristics of *scn1lab* mutants and identified a pharmacological lead from a metabolism-based small library screen, suggesting therapeutic potential for neurodevelopmental disorders such as Dravet syndrome. In addition to lower glycolytic and mitochondrial respiration rates of *scn1lab* mutants reported in Kumar *et al.* (2016), we show that mutants have impaired mitochondrial function and are also hypoglycaemic (Fig. 1). The Seahorse Extracellular Flux Analyzer is a great bioenergetics tool to measure the complete metabolic profile of live zebrafish in real time (Stackley *et al.*, 2011; Gibert *et al.*, 2013; Kumar *et al.*, 2016; Ibhazehiebo *et al.*, 2018). Although impaired bioenergetics were reported in the zebrafish model of Dravet syndrome (Kumar *et al.*, 2016), this is the first time hypoglycaemia has been identified in these mutants. Most importantly, here we tested known *pck* activators and conducted behaviour and metabolic assays to identify the activators of gluconeogenesis that could improve both convulsive seizure-like swim behaviour and impaired metabolism in larval *scn1lab* mutants. Based on our screen, we discovered a Translocator protein (TSPO) ligand, PK11195 as the lead compound and a potential therapeutic target for Dravet syndrome. The final step in this process confirmed PK11195 as the most promising drug for its anti-epileptic property using electrophysiology. According to our experimental workflow (Fig. 2) and strict criteria, only drugs that increased *pck1* or *pck2* (or both) expression more than 2-fold were included for behaviour and metabolism-based screening. The gene expression studies suggested that the vast majority of the drugs increased PEPCK-C, encoding *pck1* gene present in the cytosol but not PEPCK-M, encoding *pck2* gene present in the mitochondria (Fig. 3).

Among the validated *pck1* activators, only PK11195, AC-5216 and fluoxetine significantly suppressed seizure-like swim behaviour in *scn1lab* mutants (Fig. 3). Interestingly, in our metabolism platform, we found drugs such as PK11195, 9-*cis* retinoic acid, 8-bromo-cAMP, isoprenaline, formoterol and ractopamine could improve metabolic deficits and glucose levels (Fig. 4). The top candidate, PK11195, the ligand of the outer mitochondrial membrane was chosen due to its ability to consistently up-regulate *pck1* expression, normalize metabolic deficits, restore glucose levels and improve swim behaviour in the *scn1lab* mutant. Interestingly, several drugs with similar potential as PK11195 to improve metabolism in *scn1lab* mutants, did not suppress seizure-like swim behaviour in the mutants. A secondary electrophysiology assay using LFP recordings verified the anti-seizure properties of PK11195 and ruled out the possibility of false-positives from the locomotion assay (Fig. 6); (Baraban *et al.*, 2013; Dinday and Baraban, 2015; Griffin *et al.*, 2017, 2018, 2020). In contrast, stiripentol, an FDA-approved anti-seizure drug used in conjunction with clobazam for the treatment of patients with Dravet syndrome (Fisher, 2011) decreased swim behaviour but not *pck* expression or metabolism in *scn1lab* mutants, confirming that it targets a distinct pathway separate from PK11195. Moreover, the inability of stiripentol to inhibit metabolic deficits suggests that decreasing swim behaviour was not sufficient to account for the effects of PK11195.

PK11195 is a classical synthetic ligand of the 18-kDa TSPO, which is previously known as peripheral-type benzodiazepine receptor and is located specifically in the outer mitochondrial membrane (Braestrup and Squires, 1977; Papadopoulos *et al.*, 2006, 2007; Rupprecht *et al.*, 2010; Bhoola *et al.*, 2018). TSPO is an evolutionarily conserved and composed of five trans-membrane proteins and is widely expressed in mostly all mammalian organs (Anholt *et al.*, 1986; Antkiewicz-Michaluk *et al.*, 1988; Benavides *et al.*, 1989; Bhoola *et al.*, 2018). This mitochondrial outer membrane protein is highly expressed in the steroid-producing tissues including the microglia and reactive astrocytes present in the central nervous system (Anholt *et al.*, 1986; Gavish *et al.*, 1999; Kuhlmann and Guilarte, 2000; Casellas *et al.*, 2002; Jayakumar *et al.*, 2002; Delavoie *et al.*, 2003; Lacapere and Papadopoulos, 2003; Jordà *et al.*, 2005; Maeda *et al.*, 2007; Chen and Guilarte, 2008). Among many functions of this protein, the primary and the most well-characterized function is its involvement in neurosteroid synthesis of trans-locating cholesterol inside the mitochondria (Papadopoulos *et al.*, 2006, 2007; Rupprecht *et al.*, 2010). Interestingly, TSPO is widely studied and is of great interest because of its role as a biomarker for brain inflammation associated with microglial activation in neuropathological conditions (Chauveau *et al.*, 2008; Rupprecht *et al.*, 2010). Thus, PK11195 and other synthetic ligands were developed for diagnostic neuroimaging and as neurotherapeutics. It is a TSPO antagonist with sub-nanomolar affinity (Shah *et al.*, 1994) and is widely used for decades as a positron emission tomography radioligand to detect neuroinflammation in humans (Charbonneau *et al.*, 1986; Cagnin *et al.*, 2002; Chauveau *et al.*, 2008; Miyoshi *et al.*, 2009; Owen *et al.*, 2011; Butler *et al.*, 2013; Koepp *et al.*, 2017).The mechanism by which this drug works has been controversial, but traditionally believed to work via the peripheral-type benzodiazepine receptor. Interestingly, PK11195 imaging using tracer doses is widely used in human neurological diseases including patients with temporal lobe epilepsy as a surrogate biomarker of neuroinflammation (Kannan *et al.*, 2009; Dedeurwaerdere *et al.*, 2012; Dickstein *et al.*, 2019). Peripheral-type benzodiazepine receptor-independent actions of PK11195 at pharmacological concentrations have been linked to its anti-cancer activity which sensitizes cells to the apoptosis process via a mitochondrial pathway (Gonzalez-Polo *et al.*, 2005). Relevant to our study, PK11195 and another synthetic TSPO ligand, Ro5-4864 were unexpectedly identified in larval zebrafish by large-scale screening efforts in search of small-molecule activators of fasting metabolism (Gut *et al.*, 2013). In that study, PK11195 was shown to improve glucose tolerance in obese mice, thus providing evidence of this drug as a regulator of gluconeogenesis. It is curious that other TSPO ligands, Ro5-4864 and AC-5216, did not show similar effects as PK11195 in our study. A potential reason for this difference may be due to the underlying pharmacological mechanisms of the ligands. Although PK11195 is an antagonist, Ro5-4864 and AC-5216 are both agonists for TSPO. In fact, we found that Ro5-4864 increased swim behavior, indicative of seizure-like activity in *scn1lab* larvae (Fig. 3). This is also in agreement with another study which showed opposite effects of Ro5-4864 and PK11195 on audiogenic seizures in mice (Benavides *et al.*, 1984).These authors showed that Ro5-4864 and PK11195 increased and decreased audiogenic seizures, respectively. However, another selective TSPO ligand, AC-5216, has been shown to exert rapid anxiolytic effects and thus providing further evidence of therapeutic potential of these outer mitochondrial membrane ligands (Rupprecht *et al.*, 2009). Other studies have also shown anti-anxiety and antidepressant-like activities of AC-5216 (Kita *et al.*, 2004; Qiu *et al.*, 2016). Interestingly, in addition to PK11195, we found AC-5216 and a known antidepressant drug, fluoxetine (selective serotonin re-uptake inhibitor or SSRI), to effectively reduce seizure-like swim behaviour in *scn1lab* mutants (Fig. 3). In contrast to PK11195, neither of the two drugs had any effect on metabolism-based assays and thus did not meet our criteria for further testing. Notably, PK11195 was identified as the most promising drug from our screen of 13 drugs as it was positive in all our assays (Table 1). Elevated levels of the *Tspo* gene have been linked to cancer, numerous neurological diseases and also brain injury, and thus it is a well-documented diagnostic and imaging target (Bai *et al.*, 2007; Vlodavsky and Soustiel, 2007; Rupprecht *et al.*, 2010; Buck *et al.*, 2011). Our study provides novel findings of higher mRNA levels of *Tspo* in the *scn1lab* mutants compared to the wild-type controls (Fig. 5). This suggests that *scn1lab* mutants have elevated neuroinflammation potentially associated with microglial activation. Although we did not see a significant change with PK11195 treatment, the decreased trend in *Tspo* expression looks promising (Fig. 5). However, to conclude if PK11195 is acting as an antagonist needs further exploration.

Most importantly, our study identified a therapeutic potential of the TSPO ligand, PK11195, as it was the only drug that reversed metabolic deficits and inhibited electrographic seizures in the *scn1lab* mutants at low micro-molar concentrations. It is interesting to note in the 2013 study (Gut *et al.*, 2013), glucose control was achieved by PK11195 concomitant with an up-regulation of *pck1* in a model of pre-diabetes and obesity typically associated with hyperglycaemia. In contrast, our results indicated that PK11195 treatment elevated energetic pathways (ECAR and OCR) as well as glucose levels in the *scn1lab* mutants to wild-type levels. Taken together, this suggests that PK11195 being a gluconeogenesis modulator may serve as a glucose ‘sensor’ and ‘normalize’ impaired glucose metabolism and consequently OCR. Our data suggest that PK11195 treatment selectively ‘senses’ glucose and metabolic dys-regulation in the *scn1lab* mutants, and modulates the deficits via *pck1* expression. Interesting, in wild-type larvae, we did not observe significant *pck1* or *pck2* up-regulation when treated with the three TSPO ligands, PK11195, Ro5-4864 and AC-5216 (Supplementary Fig. 1). This finding validates our rationale for hypothesizing PK11195 as a ‘sensor’. As no abnormality was detected in wild-type larvae, PK11195 treatment did not have any effect on up-regulation of gluconeogenesis.

Thus, targeting glucose metabolism via gluconeogenesis may provide a novel therapeutic strategy to attenuate seizure activity. Although it is unclear how metabolism-based therapies exert anti-seizure effects, our study suggests one possible mechanism via ‘normalizing’ glucose by gluconeogenesis which is independent of glycolysis. The best evidence implicating the role of such a metabolic switch to achieve seizure control is use of the KD which also diverts fuels from glycolysis (Kinsman *et al.*, 1992; Kang *et al.*, 2005; Kossoff *et al.*, 2009; Nabbout *et al.*, 2011; Veggiotti *et al.*, 2011; Laux and Blackford, 2013; Gano *et al.*, 2014; Dressler *et al.*, 2015). In accordance with this, ketogenic substrates have also been shown to rescue impaired metabolism and attenuate seizures in *scn1lab* mutants (Baraban *et al.*, 2013; Kumar *et al.*, 2016). However, additional underlying mechanisms cannot be ruled out and need further exploration.

Our study did not identify the target tissue or organelle underlying the benefits of PK11195 in the mutant zebrafish. Localization of *pck1* is predominantly in the liver; although brain *pck1* expression has been noted where its functions may be related to glyceroneogenesis (Owen *et al.*, 2002; Cadoudal *et al.*, 2008). Since we assayed whole zebrafish larvae, it is likely that lower glucose levels and bioenergetics in *scn1lab* mutants were reflective of the yolk sac which serves as the major source of glucose for this larval stage rather than brain levels. The observation that metabolic deficits and seizures in *scn1lab* mutants can be controlled via gluconeogenesis is intriguing regardless of whether the brain and/or liver were targeted by PK11195 action. The role of *pck1* was further suggested by the demonstration that treatment of wild-type larvae with 3-MPA, a well-known gluconeogenesis inhibitor caused metabolic and behaviour defects similar to those observed in *scn1lab* mutants. Furthermore, PK11195 treatment rescued decreased glucose levels in the 3-MPA-treated zebrafish larvae in a manner similar to *scn1lab* mutants (Fig. 5). Moreover, treatment with known gluconeogenesis regulators, isoprenaline and formoterol, is also known to increase glucose levels (Gut *et al.*, 2013) similar to what we observe in our screen (Fig. 4).

It is noteworthy that bioenergetic platforms such as the one we utilized are increasingly used in the literature to identify metabolism-based anti-seizure drugs. Using a similar strategy, a recent study (Ibhazehiebo *et al.*, 2018) identified an approved anti-cancer drug, vorinostat, which improved mitochondrial bioenergenics and seizures in a potassium channel gene knockdown epilepsy model (*kcn1a*). Interestingly, they observed opposite OCR effects in the *kcna1*-morpholino zebrafish larval model in contrast with the *scn1lab* mutants (Fig. 1). This may be related to differences in the targeted ion channels, method used to target the ion channels i.e. acute seizures in *kcn1a* morphants similar to chemically induced seizures induced by pentylenetetrazole versus chronic seizures in *scn1lab* mutants generated by a chemical mutagenesis screen, or differences in the seizure burden in the two models. In contrast with the *kcna1* epilepsy model, a striking characteristic of the *scn1lab* mutant is the severe and relentless seizures which may cause shunting of glucose from the embryo sac and periphery to the brain which then utilizes it as an energy source for seizures. This could result in the depletion of peripheral and sac glucose, decreased ECAR and as a consequence decreased OCR in *scn1lab* mutants.

## Conclusion

In summary, we show that correcting gluconeogenesis, a pathway biochemically distinct from glycolysis, is able to rescue metabolic defects and seizures in a pre-clinical model of Dravet syndrome. This suggests that targeting energy-producing pathways is a viable and novel therapeutic avenue for the treatment of genetic neurodevelopmental disorders such as Dravet syndrome. Although our study focussed on Dravet syndrome, a rare genetic epilepsy, our findings may apply to other forms of epilepsy.

## Supplementary material

Supplementary material is available at *Brain Communications* online.

## Supplementary Material

fcab004_Supplementary_Data

## Abbreviations

DMSO =: dimethylsulphoxide
dpf =: days post-fertilization
ECAR =: Extracellular Acidification Rate
FCCP =: trifluoromethoxy carbonylcyanide phenylhydrazone
KD =: ketogenic diets
LFP =: local field potential
OCR =: oxygen consumption rate
PEPCK =: phosphoenolpyruvate carboxykinase
SSRI =: selective serotonin reuptake inhibitor
TSPO =: Translocator protein
