# Supplementary material for: Enhancing glucose metabolism via gluconeogenesis is therapeutic in a zebrafish model of Dravet syndrome
Source: Brain Commun. 2021 Jan 25;3(1):fcab004. doi: 10.1093/braincomms/fcab004 (PMC8023476; doi:10.1093/braincomms/fcab004)
Supplement: fcab004_Supplementary_Data [file fcab004_Supplementary_Data.pdf]

Supplementary Fig 1

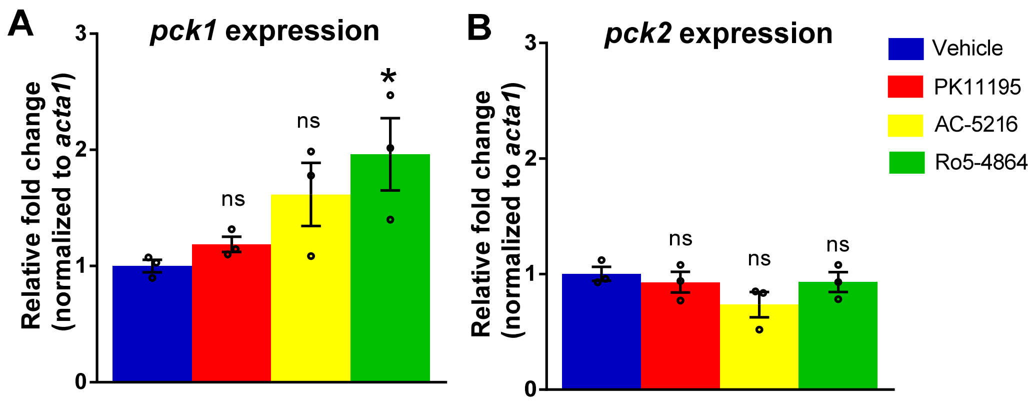

Figure 1. The expression levels of *pck* genes in wild-type larvae. (A) The bar graph represents qPCR data with relative fold change of *pck1* gene in 3-hr drug treated wild-type larvae. No significant upregulation of *pck1* was observed with PK11195 and AC-5216, whereas a less than two-fold upregulation was observed with Ro5-4864. **(B)** The bar graph represents qPCR data with relative fold change of *pck2* gene in 3-hr drug treated wild-type larvae. No significant upregulation of *pck2* was observed. Data for A and B were normalized to the housekeeping gene *acta1* and presented as mean ± S.E.M. Statistics were performed by one-way ANOVA followed by Dunnett's multiple comparison test with significance taken as $*p < 0.05$. Values represent averages from n = 3, where each sample represents 10-12 pooled 6 dpf vehicle-treated wild-type larvae or drug-treated wild-type larvae .
